# High-resolution single-particle cryo-EM of samples vitrified in boiling nitro­gen

**DOI:** 10.1107/S2052252521008095

**Published:** 2021-09-07

**Authors:** Tyler Engstrom, Jonathan A. Clinger, Katherine A. Spoth, Oliver B. Clarke, David S. Closs, Richard Jayne, Benjamin A. Apker, Robert E. Thorne

**Affiliations:** a MiTeGen, LLC, PO Box 3867, Ithaca, NY 14850-3867, USA; b Physics Department, Cornell University, Ithaca, NY 14853, USA; c Cornell Center for Materials Research, Cornell University, Ithaca, NY 14853, USA; d Department of Physiology and Cellular Biophysics, Columbia University, New York, NY 10032, USA; e Department of Anesthesiology, Columbia University, New York, NY 10032, USA

**Keywords:** cryoelectron microscopy, cryocooling, vitrification

## Abstract

Based on work by Dubochet and others in the 1980s and 1990s, samples for single-particle cryo-electron microscopy (cryo-EM) have been vitrified using ethane, propane or ethane/propane mixtures. These liquid cryogens have a large difference between their melting and boiling temperatures and so can absorb substantial heat without formation of an insulating vapor layer adjacent to a cooling sample. However, ethane and propane are flammable, they must be liquified in liquid nitro­gen immediately before cryo-EM sample preparation, and cryocooled samples must be transferred to liquid nitro­gen for storage, complicating workflows and increasing the chance of sample damage during handling. Experiments over the last 15 years have shown that cooling rates required to vitrify pure water are only ∼250 000 K s^−1^, at the low end of earlier estimates, and that the dominant factor that has limited cooling rates of small samples in liquid nitro­gen is sample precooling in cold gas present above the liquid cryogen surface, not the Leidenfrost effect. Using an automated cryocooling instrument developed for cryocrystallography that combines high plunge speeds with efficient removal of cold gas, we show that single-particle cryo-EM samples on commercial grids can be routinely vitrified using only boiling nitro­gen and obtain apoferritin datasets and refined structures with 2.65 Å resolution. The use of liquid nitro­gen as the primary coolant may allow manual and automated workflows to be simplified and may reduce sample stresses that contribute to beam-induced motion.

## Abstract 2

Samples for single-particle cryo-electron microscopy can be routinely vitrified using only liquid nitro­gen at its boiling temperature, allowing sample handling workflows to be simplified and sample stresses that contribute to beam-induced motion to be reduced.

## 1. Introduction   

Single-particle cryo-electron microscopy (cryo-EM) (Frank, 2002 ▸) has emerged as a powerful approach to obtaining near atomic resolution structures of large biomolecular complexes, membrane proteins, and other targets of major scientific, pharmaceutical and biotechnological interest (Cheng, 2015 ▸, 2018 ▸; Glaeser, 2016*b* ▸, 2019 ▸; Vinothkumar & Henderson, 2016 ▸; Lyumkis, 2019 ▸). Development of high-efficiency, high frame rate direct electron detectors (Faruqi & McMullan, 2018 ▸), algorithms for correcting acquired movies for electron-beam-induced motion (Zheng *et al.*, 2017 ▸), and computational tools for classifying and averaging 10^5^–10^6^ molecular images have dramatically increased achievable resolution and throughput. Major investments in new cryo-EM facilities and development of easy-to-use software (Punjani *et al.*, 2017 ▸; Zivanov *et al.*, 2018 ▸) have greatly expanded access, especially to non-experts. Unlike X-ray crystallography, cryo-EM requires only a small amount of biomolecular sample dispersed in solution. It allows the structural study of systems that have been intractable to crystallization and is becoming a go-to method for initial attempts at structure determination.

As in cryo-crystallography, the key challenges in single-particle cryo-EM are associated with sample preparation and handling. The basic principles and methods in current use were developed in the 1980s (Dubochet *et al.*, 1988 ▸), and many recent advances in sample preparation technology are rooted in ideas and methods developed at that time. Biomolecule samples must be expressed, isolated and purified. Cryoprotectant-free buffer containing ∼1 mg ml^−1^ of the biomolecule of interest is dispensed onto a glow-discharge cleaned and charged, 10–50 nm-thick carbon or gold ‘foil’ supported by a 200–400 mesh copper or gold grid. Excess sample is removed by blotting and evaporation, with a target thickness of a few times the biomolecular diameter or ∼10–100 nm to maximize image signal-to-noise while minimizing preferential biomolecular orientation by interaction with interfaces. To obtain vitrified buffer for the best imaging, the sample-containing grid is plunged at 1–2 m s^−1^ into liquid ethane at *T* ≃ 90 K (produced by cooling ethane gas in a liquid-nitro­gen-cooled cup). The sample is then transferred from ethane to liquid nitro­gen (LN_2_), loaded into grid boxes, transferred to additional containers and finally to a storage Dewar. Samples are then removed from the storage Dewar and grid boxes, and loaded into a cold microscope stage. Alternatively, samples may be ‘clipped’ before or after cooling for eventual loading into a cold sample cassette compatible with automated grid handlers. The stage or cassette is then loaded into the TEM.

These procedures are fraught with difficulty. Grids and especially foils are fragile and are routinely bent, torn and otherwise damaged during handling. Sample dispensing, blotting and evaporation are imprecise, and the final sample film thicknesses is poorly controlled. Biomolecules may accumulate at interfaces where they may have preferential orientation or undergo denaturation (Glaeser, 2016*a* ▸; D’Imprima *et al.*, 2019 ▸). Plunge-cooled samples can develop significant crystalline ice and are further contaminated by ice that forms on the liquid ethane, liquid nitro­gen and other cold surfaces exposed to air. Previous generation instruments in wide use for sample blotting and plunge-cooling, notably the Vitrobot from FEI, the Cryoplunge from Gatan and the EM GP from Leica, do not fully address these challenges. A new generation of much more complex instruments, such as the Chameleon from SPT LabTech (Dandey *et al.*, 2018 ▸) and the VitroJet under development at the University of Maastricht (Ravelli *et al.*, 2020 ▸), further automate the sample preparation process, combining sample dispensing, blotting/wicking, plunge-cooling and transfer to grid boxes.

A factor in the complexity of both manual sample preparation procedures and automated dispensing/blotting/cooling instruments is the required use of different liquid cryogens for initial sample cooling and for subsequent storage, transport and measurement. Since Dubochet’s work in the 1980s, nearly all cryo-EM sample-cooling devices have used liquid ethane held just above its melting point (90.3 K) for initially cooling and vitrifying samples; propane, ethane/propane and related hydro­carbons with large differences between their melting and boiling temperatures have also been used. In his 1988 review (Dubochet *et al.*, 1988 ▸), Dubochet stated that plunging water films on cryo-EM grids in boiling LN_2_ always yielded films of hexagonal ice, and that he was aware of only one successful use of ‘slushed’ N_2_ held at its freezing point, but had not been able to reproduce it. These observations, data suggesting cooling rates in LN_2_ as much as 50× lower than in ethane due to film boiling at the sample surface (Ryan *et al.*, 1987 ▸) and success using ethane appear to have largely ended serious inquiry into the physics of cryo-EM sample cooling.

Here we show that samples for single-particle cryo-EM can be routinely vitrified on commercial grids using only boiling liquid nitro­gen. As a demonstration, we obtain reconstructions of apoferritin using an FEI Arctica microscope to 2.64 Å resolution and observe beam-induced motion comparable to or less than that obtained when samples are cooled in ethane. An all-LN_2_ cold chain can simplifiy sample preparation workflows and design of automated instruments that eliminate manual sample handling after sample deposition. Cooling in LN_2_ may also reduce stresses that contribute to beam-induced sample motion.

## 2. Key principles in cooling and vitrification of cryo-EM samples   

### 2.1. Cooling rates required for vitrification of cryo-EM samples are below 10^6^ K s^−1^   

Critical cooling rates (CCRs) – the minimum cooling rates required for sample vitrification – depend on the maximum tolerable or detectable ice fraction in otherwise vitrified samples (Berejnov *et al.*, 2006 ▸). For pure water, CCR estimates ranged from 10^5^–10^6^ K s^−1^ (Brüggeller & Mayer, 1980 ▸; Dubochet *et al.*, 1988 ▸; Mayer, 1985 ▸; Bald, 1985 ▸, 1986 ▸) to 10^7^ K s^−1^ (Uhlmann, 1972 ▸) to as high as 10^12^ K s^−1^ (Muller *et al.*, 1993 ▸), with ∼10^6^–10^7^ K s^−1^ typical in the cryo-EM literature. Extrapolation of measurements of CCR versus solute concentration for diverse solutes to zero concentration established a value of ∼250 000 K s^−1^ (Warkentin *et al.*, 2013 ▸) for a crystalline ice fraction determined by X-ray methods below ∼1% (Berejnov *et al.*, 2006 ▸; Meisburger *et al.*, 2013 ▸). Ice nucleation rates in pure water increase rapidly near the homogeneous nucleation temperature *T*_h_ ≃ 235 K and remain large on further cooling (Manka *et al.*, 2012 ▸) before dropping as the glass transition temperature *T*_g_ ≃ 136 K is approached. Ice growth velocities are largest just below 260 K and then drop on further cooling (Xu *et al.*, 2016 ▸; Montero De Hijes *et al.*, 2019 ▸) to ∼10^−2^ of their peak value at 215 K and 10^−5^ of their peak value at 185 K. Ice formation in aqueous solutions at large cooling rates occurs at large supercoolings and is homogeneous-nucleation limited (Warkentin *et al.*, 2013 ▸), and ice fractions should roughly scale with cooling time in this regime. Reducing ice fractions from 10^−2^ to 10^−6^ then requires a cooling rate 10^4^ times larger.

Fortunately, this is not necessary (Wieferig *et al.*, 2021 ▸). Crudely, high-resolution particle imaging is possible as long as the volume fraction of ice relative to the biomolecule is small within the sample so that, for most particles, there is no visible or strongly diffracting ice within the volume around a particle that contributes to the particle image; similarly, proximity of neighbors in the dense arrays of randomly oriented biomolecules often observed in cryo-EM images does not prevent high-resolution reconstructions. Warming cryo-EM samples to 160 K to release cooling-induced stress causes substantial recrystallization in an initially vitrified sample but has no significant deleterious effect on biomolecule imaging (Wieferig *et al.*, 2021 ▸; see also Cyrklaff & Kühlbrandt, 1994 ▸).

CCRs decrease exponentially with solute concentration (Warkentin *et al.*, 2013 ▸), but solutes decrease electron density and EM contrast (Tyree *et al.*, 2018 ▸). CCRs are ∼220 000 K s^−1^ for a cryo-EM buffer with ∼0.5% *w*/*v* salt concentration (Warkentin *et al.*, 2013 ▸).

### 2.2. Cooling rates achieved in current cryo-EM practice are far below theoretical limits   

Despite using liquid ethane, one of the most effective liquid cryogens, and despite modest cooling rates required to vitrify water, samples for single-particle cryo-EM can develop substantial areas of crystalline ice during cooling. For a thin-film sample comprised of 50 nm of water on 50 nm of gold or 12 nm of carbon and plunged edge-on at 2 m s^−1^ into liquid ethane at ∼90 K, an approximate analytic analysis of heat transfer [based on the work by Kriminski *et al.* (2003 ▸) and references therein] predicts cooling rates on the order of 10^7^ K s^−1^, and that cooling below the glass transition *T*_g_ ≃ 136 K of water should occur over a distance of ∼30 µm. For film-boiling LN_2_ at 77 K, the predicted cooling rate is ∼10^6^ K s^−1^ and the cooling distance is ∼300 µm. For a 30 µm-diameter water sample plunged at 2 m s^−1^, the predicted cooling rate in liquid ethane is ∼300 000 K s^−1^ (Kriminski *et al.*, 2003 ▸), comparable to the cooling rate measured using a 30 µm bead thermocouple (Costello, 2006 ▸). But when appreciable crystalline ice forms in a single-particle cryo-EM sample, the cooling rate must be below ∼200 000 K s^−1^. Why might cooling rates be so low?

### 2.3. Average cooling rates can be limited by precooling in cold gas above liquid cryogens   

Cold gas above a liquid cryogen precools samples as they are plunged through it (Ryan, 1992 ▸). For plunge speeds of ∼1 m s^−1^, a cold gas layer only ∼2 cm thick is sufficient to dominate cooling of protein crystallography samples <0.1 µl in size (Warkentin *et al.*, 2006 ▸; Berejnov *et al.*, 2006 ▸). Both commercial and home-built cryo-EM plunge-cooling instruments plunge the sample into a small ethane-filled cup surrounded by a larger LN_2_-filled container (Fig. S1 of the supporting information). The ethane level is typically at a millimetre or more below the top of its cup. The top of the ethane cup may be just above or well below the top of the LN_2_ container. A layer of cold gas at least a few millimetres thick – often revealed via water droplet and ice crystal ‘fog’ – forms along the sample plunge path to the liquid ethane. With a predicted cooling rate for the sample + foil in dry N_2_ gas at ∼100 K of ∼200,000 K s^−1^, the sample need only travel ∼1.4 mm – half the grid diameter – through this gas before it has vitrified, a distance comparable to or smaller than any plausible cold gas layer thickness. The presence of (much larger thermal mass) 10–20 µm-thick grid bars reduces cooling rates nearby, but cooling rates of sample + foil near the center of grid openings may be of this order (Thorne, 2020 ▸). Consequently, cooling of the sample and foil between grid bars may largely occur in the cold gas, before the sample reaches the ethane. The thickness of the cold gas layer and the fraction of cooling it provides depends on, for example, ethane and nitro­gen fill levels, time since filling, and chance breezes, and may contribute to the variability of observed cooling outcomes.

As discussed in Section 5.2, precooling in cold gas is generally much more severe when using boiling LN_2_ than when using ethane held just above its melting temperature. Confusion about the importance of this precooling when thermocouples are plunged into LN_2_, and when samples much thinner than available thermocouples are cooled, appears to have caused the cooling potential of LN_2_ relative to ethane for thin samples to have been underestimated.

## 3. Experimental methods   

### 3.1. Sample grids   

Three different types of grids (*i.e.* grid + sample support foil) were used for single-particle imaging experiments. The first (grid Type A) was a commercial UltrAuFoil R1.2/1.3 300 mesh grid with a Au grid and Au foil, from Quantifoil (Jena, Germany). The second (grid Type B) was an in-house developed 300 mesh Cu grid/Au foil prototype. The third grid type (Type C) combined a 300 mesh EMS Au grid (Hatfield, PA, USA) and an in-house-made Au foil. Details of grid and foil fabrication are given in the supporting information.

No more than 30 min prior to sample application, grids were rendered hydro­philic in a Harrick Plasma PDC-32G system, using 600 mTorr of air plasma at the ‘high RF’ setting (18 W of coil power) for 2 min.

### 3.2. Sample cryocooling   

Samples were cryocooled using the MiTeGen NANUQ automated liquid nitro­gen-based cryocooler for cryocrystallography (Figs. S2 and S3), which is based on insights into the physics of cryocooling described by Kriminski *et al.* (2003 ▸) and Warkentin *et al.* (2006 ▸). Briefly, NANUQ consists of a high-speed (2 m s^−1^) vertical sample translation stage, a gas management manifold containing a plunge bore, an insulated tank filled with boiling LN_2_ beneath the gas manifold, an automated sample carousel in the tank and an LN_2_ level control system. The gas management manifold (Fig. S3) uses a combination of heaters, dry room temperature N_2_ gas and suction to completely remove cold gas above the LN_2_ within the bore and to maintain the temperature within the bore above 273 K to within <50 µm of the LN_2_ surface. This ensures that nearly all sample cooling occurs once the sample enters the LN_2_. The gas management manifold also isolates all cold surfaces from ambient air to minimize or eliminate frost accumulation. The sample carousel accepts four standard 16-sample storage and shipping ‘pucks’ used in automated handling at synchrotron source beamlines and automatically positions an empty sample slot beneath the plunge path after each plunge. Using thermocouples with ∼30 µm beads, measured cooling rates in boiling nitro­gen using NANUQ are in excess of 50 000 K s^−1^.

To use NANUQ in cryocrystallography, a nylon or microfabricated ‘loop’ attached to a magnetic steel goniometer base is held using a magnetic wand. Crystals are looped or scooped out of solution onto the loop, and then the wand, base, loop and crystals are loaded on the NANUQ vertical translation stage. When the access door to the vertical stage is closed, the cold gas above the LN_2_ within the plunge bore is removed and replaced with dry ambient-temperature gas, the sample is plunged into the LN_2_, and then the sample is translated and released into the storage puck.

To use this instrument for cryocooling cryo-EM grids, two generations of prototype grid holders consisting of custom forceps attached to standard crystallography goniometer bases were fabricated. A grid was grasped by a grid holder, which in turn was held by the magnetic wand, and then the grid + holder + wand were loaded on the NANUQ vertical translation stage. After cooling, samples were automatically transferred into one of two standard sample holding ‘pucks’ used in cryocrystallography: a UniPuck for the first-generation grid holder and a cryovial-containing CombiPuck for the second-generation grid holder (Fig. S4).

### 3.3. Sample preparation   

Initial experiments in summer 2020 used a protein-free 0.5% *w*/*w* NaCl solution. With a 400 mesh Quantifoil R 2/2 holey carbon grid attached to a first-prototype grid holder and wand in the NANUQ ready-to-plunge position, and with the grid in ambient air having ∼50% relative humidity (r.h.), 2 µl of solution was pipetted onto the foil side of the grid. The grid was manually blotted for 2–3 s using Whatman no. 1 paper, and then immediately plunged at 2 m s^−1^ into LN_2_ and automatically transferred into a UniPuck immersed in the LN_2_. When sample plunging was complete, the UniPuck was removed from NANUQ and transferred to an LN_2_-filled insulated container, and samples were removed from the puck one by one and released into standard cryo-EM grid boxes. These grid boxes were then transferred to an LN_2_-filled Dewar for storage and transport to the TEM.

Subsequent experiments used a solution comprised of 5 mg ml^−1^ equine spleen apoferritin (Sigma, catalog No. A-3641) in 50 m*M* HEPES buffer with pH 7.4. The initial grid position and its plunge path were humidified to 90% r.h. reducing the film evaporation velocity to ∼5 nm s^−1^, making the timing of blotting and evaporation less critical. Second-generation prototype grid holders improved ease of gripping and grid perpendicularlity to the LN_2_ surface during the plunge. A 3 µl volume of solution was pipetted onto the foil side of either a Quantifoil (Type A) or MiTeGen prototype (Type B, C) grid, and the excess was blotted by hand with Whatman no. 1 paper for between 2 and 8 s. Each grid was then immediately plunged at 2 m s^−1^ into LN_2_ at 77 K, and then automatically translated into cryovials held within a CombiPuck. After all samples in a run had been cooled, the CombiPuck was removed from the NANUQ carousel, the cryovials removed from the CombiPuck, the grid and holder removed from each cryovial using a standard wand, and the grid released into a SWISSCI cryo-EM grid box. The grid boxes were then stored in a MiTeGen cryo-EM puck, which was loaded into a Worthington CX100 shipper with a MiTeGen cryo-EM transport cane.

### 3.4. Microscope data collection   

Cryo-EM grid screening and data collection were performed using a Thermo Fisher Scientific Talos Arctica cryo-TEM operating at 200 kV and equipped with a K3 direct electron detector operating in electron counting mode and a Bioquantum energy filter (Gatan). Grid screening (but not data collection) was also performed using an FEI Tecnai 12 BioTwin TEM operating at 120 kV and equipped with a Gatan Orius 1000 dual-scan CCD detector. Both TEMs were housed at the Cornell Center for Materials Research.

### 3.5. Ice characterization   

Approximate ice thickness on the grids was determined by comparing the intensity measured with and without the 20 eV energy filter. The apparent mean free path for this microscope was measured to be 290 nm by comparing the log of the intensity ratio of unfiltered over filtered images to the absolute thickness determined by tomography (Rice *et al.*, 2018 ▸).

The absolute thickness was determined in two holes on a type B grid (sample 2) using tilt-series tomography (Fig. S5). Tilt series in 3° increments with a tilt range from −60 to +60° were collected using *SerialEM* (Mastronarde, 2005 ▸) on the Arctica at a magnification of 49k×, corresponding to a 1× binned pixel size of 1.58 Å, using a dose-symmetric scheme (Hagen *et al.*, 2017 ▸). At each tilt, a five-frame movie was collected for 0.16 s, corresponding to a 2.75 e^−^ Å^−2^ exposure per tilt and 113 e^−^ Å^−2^ total exposure. *IMOD* software was used to perform the 3D reconstruction from the tilt frames (Kremer *et al.*, 1996 ▸).

### 3.6. Single-particle cryo-EM data collection   

Two complete single-particle datasets, one on a Type A grid and another on a Type B grid, were collected on the Arctica using a 100 µm objective aperture and 20 eV energy slit, under near-identical conditions but on different days. Both datasets were collected at 63k× magnification, corresponding to 0.615 Å per 0.5×-binned pixel. 50-frame movies were collected using a total exposure of 55 e^−^ Å^−2^ and exposure time of 3 s. The exposure rate at the detector over vacuum was 28 e^−^ pixel^−1^ s^−1^. Data acquisition was automated using the *SerialEM* software (Mastronarde, 2005 ▸). Beam-image shift was used to collect one movie per hole in a 3 × 3 hole pattern, with beam tilt correction applied in *SerialEM*. High-resolution imaging data from ∼50–120 foil holes, sufficient for beam-induced-motion analysis, was collected from four additional grids.

### 3.7. Data processing and single-particle reconstruction   

Single-particle datasets were processed using *cryoSPARC* (Punjani *et al.*, 2017 ▸). A dataset for sample 1 on an UltrAuFoil grid consisted of 159 micrographs, with a measured defocus range from 0.6 to 1.8 µm. After patch-based motion correction and CTF estimation using Patch CTF, micrographs were manually inspected and 136 were selected for use. In total, 200 manually picked particles from 20 micrographs were classified into 4 templates and used to pick 129 019 particles from the micrographs. Particles were extracted at 0.615 Å per pixel using a box size of 416 pixels. 2D classification was used to remove junk, leaving 95 834 particles. After *ab initio* reconstruction and heterogeneous 3D classification into five classes, the highest-resolution class was kept, consisting of 59 824 particles. This class was subjected to homogeneous refinement with octahedral symmetry enforced and both global and local CTF refinement, resulting in a 2.64 Å map.

A dataset collected for sample 2 on a prototype grid consisted of 174 total micrographs. Measured defocus ranged from 0.5 to 1.7 µm. Micrographs were patch-motion corrected and patch-CTF corrected. After manual inspection, 122 micrographs were selected for continued processing. 1163 particles were blob-picked from four micrographs and 2D classified into five classes. Two classes were used for template picking from all micrographs, with 109 901 total particles picked. Particles were extracted at 0.615 Å per pixel in 416 pixel boxes. 2D classification was used to remove junk particles, with 84 369 particles kept. Two rounds of 3D classification were performed, using 5 and 3 classes; the highest-resolution class selected for further processing had 22 027 particles. Homogeneous refinement of this class with octahedral symmetry enforced and global CTF refinement produced a 2.85 Å map.

### 3.8. Model building and refinement   

Model building and refinement were performed using each map (individually). A high-resolution crystal structure of apoferritin (PDB entry 2w0o; De Val *et al.*, 2012 ▸) was used as the starting model with all non-protein residues removed. This model was placed in the map using *phenix.dock_in_map* (Liebschner *et al.*, 2019 ▸) and refined in *Coot* (Emsley *et al.*, 2010 ▸) and using *phenix.real_space_refine* (Liebschner *et al.*, 2019 ▸). The final refinement used one half map, and validation was carried out against the other half map. *ChimeraX* (Goddard *et al.*, 2018 ▸) was used for visualization of the model during refinement and *PyMOL* (Schrodinger) was used to make figures. Statistics for the two models are given in Tables 1 and S1 of the supporting information.

### 3.9. Analysis of beam-induced motion   

In-plane particle trajectories were determined using a local motion job in *cryoSPARC* (Punjani *et al.*, 2017 ▸) on all the template-picked particles in each dataset (∼100 000 particles for samples 1, 2 and 7 and ∼50 000 particles for the other samples). Initial motion correction for each micrograph was performed using the *cryoSPARC* patch-based motion correction job; this corrects for both rigid-body motion (*i.e.* stage drift) and local beam-induced motion. The local per-particle motion was then analyzed by computing the RMS particle displacement (in-plane component) as a function of fluence, where the mean is taken over all picked particles.

## 4. Results   

### 4.1. Vitrification of a 0.5% NaCl solution using boiling liquid nitro­gen   

**Figure 1:**
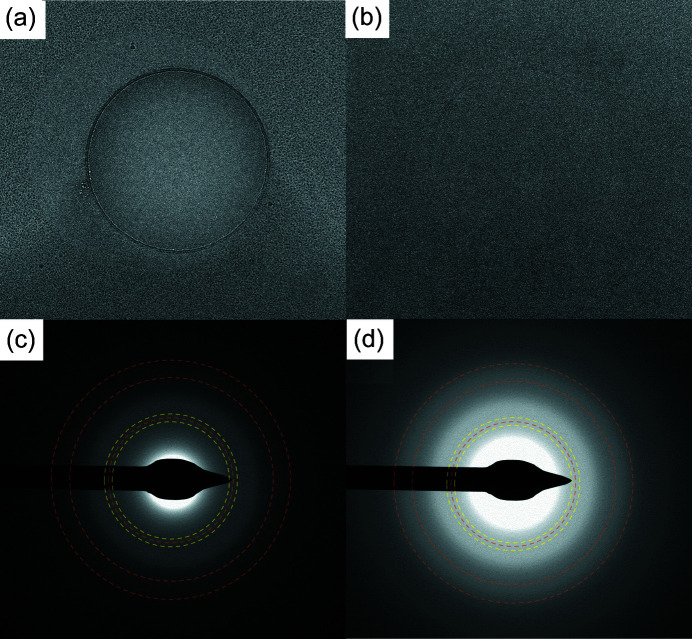
(*a*) and (*b*) Real space detector images and (*c*) and (*d*) corresponding diffraction mode detector images of a biomolecule-free 0.5% NaCl solution on a 400 mesh Quantifoil holey carbon grid with 2 µm holes plunge-cooled in boiling LN_2_. Both thick and thin ice were fully vitrified with no ice diffraction evident. The dashed lines in (*c*) and (*d*) indicate the expected positions of pure cubic ice diffraction (orange lines) at 1/*d* = 2.73, 4.45 and 5.22 nm^−1^ and of stacking disordered ice diffraction which, in addition to the peaks of cubic ice, typically has additional strong peaks at the hexagonal ice positions 2.57 and 2.91 nm^−1^ (yellow lines).

Fig. 1 ▸ shows cryo-EM images and diffraction patterns acquired from a 0.5% NaCl solution on a 300 mesh Quantifoil grid that was plunge-cooled in boiling LN_2_ using NANUQ. Both thick and thin ice across large areas of the grid were fully vitrified. The minimum cooling rate to vitrify a 0.5% NaCl solution is ∼200 000 K s^−1^, based on a visual ice assay that has an ice fraction detection limit of roughly 1% (Warkentin *et al.*, 2013 ▸). The complete absence of ice diffraction indicates that the cooling rate achieved was greater than 200 000 K s^−1^ and/or that some concentration of the salt occurred due to evaporation during blotting, lowering the minimum cooling rate for vitrification.

In these initial experiments using first-prototype grid holders, the main factor (aside from excessive sample thickness) preventing vitrification was deviation of the grid plane from absolute perpedicularity to the LN_2_ surface during plunging due to either improper grid gripping or slight grid bending from mishandling (Passmore & Russo, 2016 ▸). Deviations of 1–2° were sufficient to cause large grid bending during travel through the LN_2_, residual permanent deformation (as determined by inspecting grids through the cryovials after plunging) and frequent foil damage. These likely reduced cooling efficiency. Grid bending during plunging should grow with distance traveled through liquid cryogen. In NANUQ, the plunge distance is >3 cm, much larger than in typical ethane-based cryo-EM plunge-coolers. The resulting transient bending sometimes exceeded the elastic limit of the grid, causing permanent deformations that provided direct evidence for plunge-induced bending that otherwise would be difficult to detect.

### 4.2. Vitrification of apoferritin solutions using boiling liquid nitro­gen   

**Figure 2:**
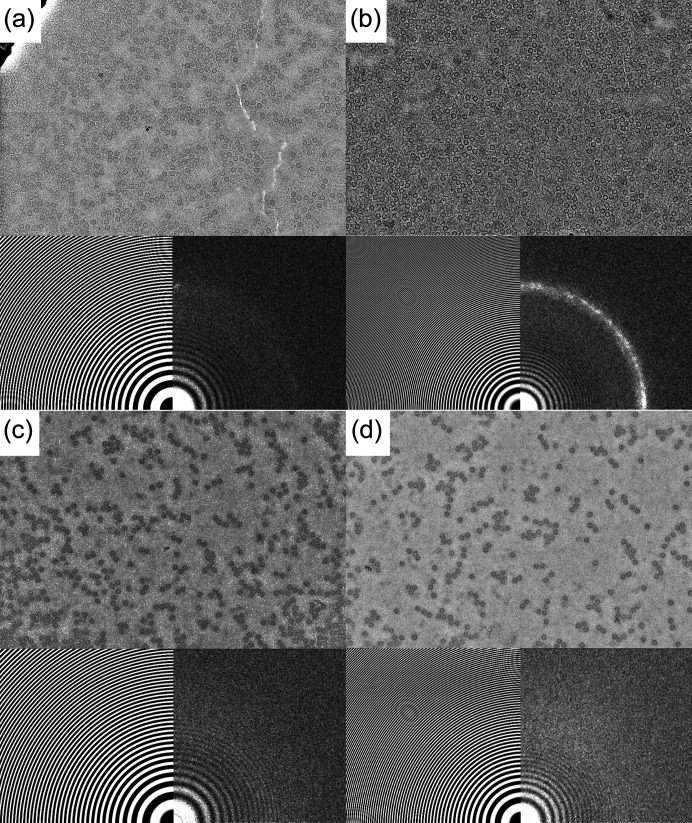
Sample real space detector images and corresponding image CTFs and FFTs of 5 mg ml^−1^ apoferritin solutions that were dispensed and blotted on (*a*) and (*b*) a Quantifoil UltraAuFoil grid (sample 1) and (*c*) and (*d*) a prototype grid (sample 2), both having gold foils with 1.2 µm holes, and then plunge-cooled in boiling LN_2_. Sample film thicknesses and areal particle densities were typically larger for sample 1 than sample 2. The majority of hole images and FFTs for sample 1 showed evidence of small amounts of crystalline ice, whereas nearly all hole images for sample 2 were fully vitrified.

After initial debugging trials to improve grid gripping and determine appropriate blotting times when operating in a 90% r.h. environment, two batches of grids were prepared using the 5 mg ml^−1^ apoferritin solution. In the first batch, 16 grids were plunge-cooled, 12 were screened and of these 3 had good ice suitable for high-resolution single-particle imaging. In the second batch, 16 grids were plunge-cooled, 12 were screened and 8 had good ice. Good ice was achieved reproducibly using both the Quantifoil grids and the MiTeGen grid prototypes. ‘Good ice’ here means that there were many grid squares with little or no visible crystalline ice and little or no evidence of ice in image FFTs or diffraction patterns (Figs. 2 ▸ and 4). Thickness estimates of ‘good’ ice on five grids, obtained by comparing intensities with and without the energy slit (Section 3.5) ranged from 10 to 50 nm.

These results establish that vitrification of cryo-EM samples using boiling LN_2_ can be routinely achieved. The most important factors affecting success are blotting time/final sample film thickness and grid flatness and orientation during plunging.

### 4.3. High-resolution single-particle imaging and reconstructions   

When sample films of appropriate thickness were generated, high-resolution images of apoferritin molecules were routinely obtained (Fig. 2 ▸). Apoferritin particle densities in these images were typical of those previously reported, so effects of solute concentration due to evaporation in the 90% r.h. atmosphere on vitrification were likely comparable to those in previous ethane-cooled samples (Section 5.2).

Two of the screened samples, sample 1 on a Quantifoil grid and sample 2 on a prototype grid, were selected for collection of datasets sufficient for single-particle reconstructions. For sample 1, FFTs of roughly 57% of hole images showed near continuous or lumpy ice ‘rings’, indicating the presence of many ice grains with different sizes; 27% showed only a few bright peaks consistent with the presence of one or two large crystalline regions; 12% appeared to be completely ice-free. Those holes with the strongest ice intensity showed the largest areal particle densities, consistent with their having the largest thicknesses. Tilt series measurements on one hole with intermediate particle densities indicated a thickness ranging from ∼20 nm near the middle of the hole to ∼30–40 nm near its edge (consistent with the 35 nm foil thickness). For sample 2, FFTs of only 11% of hole images show evidence of ice, and only 5% showed strong intensity consistent with either a few or multiple ice particles. Fig. 2 ▸ shows example images and corresponding CTFs and FFTs from each of these samples. Single-particle reconstructions obtained as described in Section 3.7 using the acquired data had resolutions of 2.64 Å for sample 1 and 2.86 Å for sample 2. Even though sample 1 was incompletely vitrified, its small ice fraction had no obvious deleterious effect, as has been found elsewhere (Wieferig *et al.*, 2021 ▸).

### 4.4. Structure modeling and refinement   

**Figure 3:**
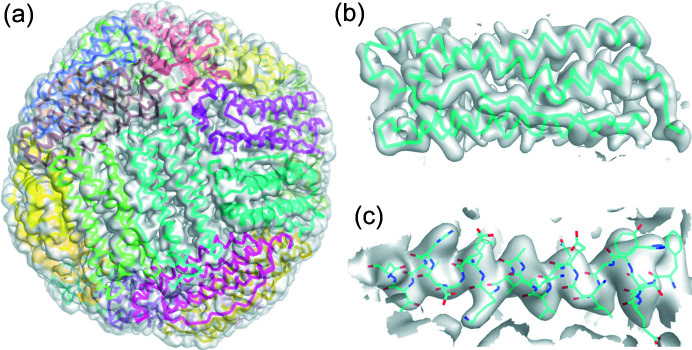
Single-particle reconstruction and refined model based on apoferritin data obtained from sample 1, which was deposited on a Quantifoil grid and plunge-cooled in boiling LN_2_. (*a*) Apoferritin model placed into surface map representation. Ribbons of apoferritin monomers colored by chain designation. (*b*) Single monomer of apoferritin showing map-monomer fit. (*c*) Apoferritin helix comprised of residues 132–154 demonstrating the sidechain fit. Maps in (*a*)–(*c*) are contoured at 1σ. The Fourier shell correlation (FSC) plot is shown in Fig. S7(*a*).

Model building and refinement based on the 2.64 Å cryo-EM apoferritin reconstruction from sample 1 gave a final model with good statistics and no artifacts (Table 1 ▸). The model (Fig. 3 ▸) shows no deviations from previous apoferritin cryo-EM structures in this resolution range, as expected considering the quality of the upstream data. The model and statistics obtained using the 2.86 Å reconstruction from sample 2 are given in Fig. S6 and Table S1, respectively.

### 4.5. Analysis of beam-induced motion   

**Figure 4:**
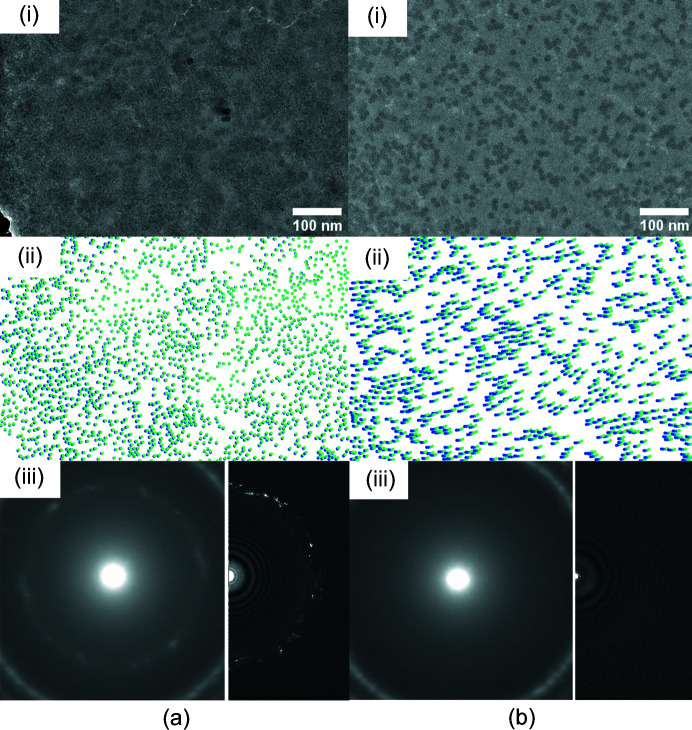
Beam-induced motion, sample thickness and ice for (*a*) sample 3 and (*b*) sample 4. Shown are (i) an image of a foil hole at a fluence of 1.00 e^−^ Å^−2^; (ii) particle positions measured in the first and fifth frames corresponding to fluences of 0.55 and 5.5 e^−^ Å^−2^, respectively; (iii) sample film thickness map determined by comparing transmitted intensities with and without an energy slit; and (iv) diffraction mode image (left) and FFT of real space image (right).

Fig. 4 ▸ shows a composite of the sample image, particle displacements between the first frame and the fifth frame (corresponding to a fluence of 5.5 e^−^ Å^−2^), determined as described in Section 3.9 using the *cryoSPARC* local motion correction and samples at 0° tilt; a map of the particle displacements; and both a diffraction mode image and an image FFT, all for the same hole. Sample 3 was incompletely vitrified, showing a similar extent of ice in image FFTs as sample 1. Sample 4 was largely vitrified, similar to sample 2. Sample 3 shows smaller net particle motions than sample 4, despite showing a larger fraction of crystalline ice. This could be a factor contributing to the somewhat higher resolution particle reconstruction obtained using sample 3.

**Figure 5:**
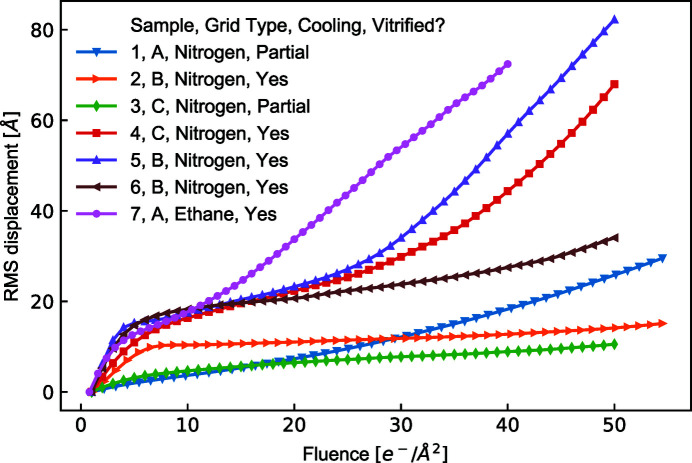
Drift-corrected RMS particle displacement versus fluence, all measured using the same cryo-TEM. Grid type A is Quantifoil UltraAuFoil 1.2/1.3, Au foil with 1.2 µm holes on Au grids; type B is prototype Au foil with 1.2 µm holes on a Cu grid; type C is prototype Au foil with 1.2 µm holes on an Au grid. Samples 1–6 were cooled in boiling LN_2_, and sample 7 was cooled in ethane using a Vitrobot Mark IV. Samples 1 and 3 were partially vitrified; most holes gave good particle images but showed local ice, confirmed by appreciable intensity at ice ring positions in image FFTs. Samples 2 and 3–7 were largely vitrified with only a small minority of frames showing evidence of ice.

Fig. 5 ▸ shows the RMS displacement associated with local (rigid-body-motion subtracted) motions versus fluence for several samples, all on Au foils with 1.2 µm-diameter holes, all measured in the same cryo-TEM and all determined as described in Section 3.9. For all samples including samples 2 and 4 showing fully or mostly vitrified ice (*i.e.* FFTs of most hole images show no ice or only weak diffuse ice rings), the displacement has a steep initial increase with fluence followed by a more gradual increase at fluences beyond ∼5 e^−^ Å^−2^, as is typically observed. The initial slopes of displacement versus fluence for these samples range from ∼2 to ∼6 Å^3^/e^−^. Sample 7 in Fig. 5 ▸, on a Quantifoil UltraAuFoil 1.2/1.3 grid, was plunge-cooled in liquid ethane using a Vitrobot Mark IV, and shows an initial slope of 3.6 Å^3^/e^−^, similar to that of the vitrified samples cooled in boiling LN_2_. These values for boiling LN_2_ and ethane-cooled samples compare with a value of ∼1.9 Å^3^/e^−^ reported for an ethane-cooled sample on a foil with 1.2 µm diameter holes (Naydenova *et al.*, 2020 ▸) and of ∼1.0–1.8 Å^3^/e^−^ reported for ethane-cooled samples on foils with 2 µm diameter holes (Wieferig *et al.*, 2021 ▸), all measured at 0° tilt. However, for LN_2_-cooled samples 1 and 3, for which FFTs and/or diffraction patterns showed significant ice intensity, the initial slopes were only 0.5 and 0.9 Å^3^/e^−^. These compare with initial slopes of ∼0.3 Å^3^/e^−^ for ethane-cooled samples on foils with 2 µm diameter holes measured after partial devitrification by transient warming (Wieferig *et al.*, 2021 ▸).

## 5. Discussion   

### 5.1. Measurement of cooling rates and the relative effectiveness of liquid cryogens   

Direct measurements of the temperature-time response of sub-100 nm-thick cryo-EM samples on 10–50 nm-thick foils has so far not been possible. Almost all measurements have used thermocouples formed from 12.5 or 25 µm-diameter wire and having junction beads of sizes between ∼25 and 75 µm. Early thermocouple measurements during plunging in iso­pentane yielded cooling rates of ∼300 000 K s^−1^ (Luyet & Gonzales, 1951 ▸), and have not been substantially improved upon in the subsequent 70 years (Costello, 2006 ▸; Ravelli *et al.*, 2020 ▸). At best, these measurements reflect cooling rates of 20–25 µm-thick grids, setting a lower bound on what may be achieved in sub-100 nm-thick cryoEM samples within grid openings under optimal cooling conditions.

Thermocouple response times may have obscured the importance of precooling of 10–100 nm-thick sample films in cold gas layers during high-speed plunges in ethane, where the cold gas layer may be only a few millimetres thick. For a sufficiently small/thin sample, cooling rates will be limited by the cold gas layer thickness and plunge speed (Warkentin *et al.*, 2006 ▸), which for a 2 mm thickness and 2 m s^−1^ plunge speed is roughly 150 000 K s^−1^.

On the other hand, cold gas layers above LN_2_ at 77 K in cryo-EM Dewars, generated by boiling as well as by conduction, convection and radiation, can be several centimetres thick, sufficient to substantially cool ∼30 µm thermocouples during ∼1 m s^−1^ plunges. This may explain reported cooling rates in boiling nitro­gen as much as a factor of 50 smaller than those obtained in ethane at 90 K (Ryan *et al.*, 1987 ▸; Ravelli *et al.*, 2020 ▸). Cooling rates measured in boiling nitro­gen with NANUQ – using a 30 µm bead, 12.5 µm lead wire thermocouple – of 50 000 K s^−1^ are within a factor of 7 of the largest cooling rates ever reported in any liquid cryogen [measured using a thermocouple with a 30 µm bead and 12.5 µm lead wires plunged at 2 m s^−1^ into liquid propane (Costello, 2006 ▸)]. The cooling rate ratio between boiling nitro­gen and ethane/propane near 90 K depends on the sample size and sample thermal conductivity, which affect the duration of film boiling at the sample surface, and on the plunge speed (Bald, 1984 ▸; Gakhar & Wiencek, 2005 ▸; Warkentin *et al.*, 2008 ▸). The cooling rate ratio for ∼10–100 nm aqueous films should be smaller than for 30 µm metal thermocouples.

The relative cooling effectiveness of ethane and nitro­gen may be affected by the behavior of the gas–liquid–grid interfaces as the grid enters the liquid cryogen. As noted in Section 2.3, at cooling rates required for vitrification and plunge speeds of 2 m s^−1^, cooling of the grid and sample film from *T*_m_ to *T*_g_ occurs over a distance comparable to or smaller than the grid diameter. When a grid is incident edge-on at high speed, the flat surface of the liquid cryogen is disrupted. The nature and extent of the disruption depends on the grid speed, profile and initial temperature and whether the liquid cryogen wets to or is repelled by the grid surface. A high-speed imaging study of grid plunging into liquid ethane (Kasas *et al.*, 2003 ▸) was interpreted as showing that a gas-filled cavity (depression) formed around the grid as it entered the ethane. Even once the grid was fully below the (average) ethane surface, their images suggested that liquid ethane did not wet the flat faces of the grid (although their resolution did not permit them to exclude formation of a small capillary meniscus between the grid and liquid ethane). Since the ethane was far below its boiling temperature and, because the grid is so thin, surface boiling is not expected, the gas in the cavity was likely drawn from gas above the original ethane surface. Studies of spheres directed at high speed into water (Marston *et al.*, 2012 ▸) show that wettability is a key factor in determining cavity formation (Duclaux *et al.*, 2007 ▸). For spheres with hydro­philic surfaces, water wets the sphere surface to some distance above the average water level as the sphere enters. For spheres with hydro­phobic surfaces, an air-filled cavity forms around the trailing edge of the sphere. The behavior of grids in liquid ethane as reported by Kasas *et al.* (2003 ▸) suggests that ethane did not readily wet the copper grids used.

In the case of cavity formation around the grid, cooling rates will initially be limited by heat transfer through gas within the cavity to the liquid cryogen, and so will be lower than if the liquid ethane were in direct contact with the grid. LN_2_ generally wets most surfaces, so cavity formation by this mechanism is not expected. But LN_2_ at 77 K boils on contact with the warm grid and so a Leidenfrost ‘cavity’ of cold gas should still form (Marston *et al.*, 2012 ▸). The existence of gas-filled cavities in both cases may reduce differences in cooling rates that would occur if ethane fully wetted grids. This discussion suggests that grid and foil surface treatments to enhance cryogen wettability may improve cooling outcomes.

### 5.2. Why is vitrification of cryo-EM samples by plunge-cooling in boiling nitro­gen possible?   

The high-resolution single-particle reconstructions pres­ented here are, to our knowledge, the first obtained using cryo-EM samples cooled using boiling nitrogen. Heat transfer to boiling nitro­gen immediately generates gas that insulates the sample from direct contact with the liquid, and even in its liquid form nitro­gen heat-transfer properties are inferior to those of ethane or propane. Given the conventional wisdom of the last four decades, why is vitrification of cryo-EM samples in boiling LN_2_ possible?

First, cooling rates required to ‘vitrify’ pure water and cryo-EM buffers are modest: only ∼250 000 K s^−1^.

Second, ‘vitrified’ samples need not be completely free of ice nuclei and nanocrystals to yield high-resolution images (Cyrklaff & Kühlbrandt, 1994 ▸; Wieferig *et al.*, 2021 ▸). Tolerable ice fractions are larger than assumed in some previous estimates of minimum cooling rates required for vitrification, and so required cooling rates are smaller.

Third, the concentration of all solutes including buffer components and biomolecules in cryo-EM samples is increased by evaporation and by trapping at air-buffer interfaces between initial deposition through blotting to cooling, even in the 90–95% r.h. environments achieved under the most favorable circumstances. The final concentrations may sometimes be large enough (*e.g.* when the particles form densely packed semi-regular arrays) and the ‘free’ volume fraction of water (*i.e.* water not involved in hydration of solutes) small enough that cooling rates required for vitrification may be substantially reduced (Moreau *et al.*, 2019 ▸).

Finally, liquid nitro­gen at its 77 K boiling temperature is a much better coolant of small samples than suggested by previous experiments, and gives cooling rates that are, at most, only several times smaller than those in ethane at ∼90 K.

### 5.3. Beam-induced motion and choice of liquid cryogen   

As discussed by Thorne (2020 ▸), the initial rapid beam-induced motion that limits resolution in cryo-EM is likely due to compressive stress initially present in the plunge-cooled sample, primarily arising from transient differences in contraction of the support foil and grid due to their different cooling rates during plunge-cooling. The grid bars have a large thickness (10–25 µm) and thermal mass per unit area and cool relatively slowly – at a rate that may be comparable to that of 30 µm thermocouple junctions used for cooling rate measurements. The sample support foil and sample film are much thinner (∼10–100 nm), have much smaller thermal mass per unit area and conduct little heat from the grid bars, so the foil + sample in the middle of the grid openings cool faster than the grid. Consequently, substantial transient temperature differences between the foil + sample and grid bars must develop during cooling, generating transient tensile stress in the foil. When the sample vitrifies at its *T*_g_ (∼136 K) it does so on/in a foil that is under tensile stress. The tensile foil stress is released as the grid bars cool towards the liquid cryogen temperature, placing the sample within the foil holes under compressive stress. Radiation-induced creep (Bullough & Wood, 1980 ▸; Shibata, 2013 ▸) in the presence of this compressive stress then causes doming motion of the sample – the dominant component of observed rapid initial beam-induced motion.

This hypothesis for the origin of beam-induced motion is consistent with several pieces of evidence. At low total doses where motion is most rapid with dose, the RMS magnitude of particle motion within a given foil hole is (to within experimental uncertainties) proportional to the diameter of the hole (Naydenova *et al.*, 2020 ▸; see Fig. S8), as predicted by Thorne (2020 ▸): the doming amplitude *h* ∝ *a ε*, where *a* is the hole diameter and ε is the (dimensionless) foil strain released between when the sample initially vitrifies (on the tensile-stressed foil) and when the grid and foil have both cooled to the temperature of the liquid cryogen. The overall magnitude of the observed motion is consistent with rough estimates of grid-foil temperature differences during cooling (Thorne, 2020 ▸). Doming motion is reduced if the liquid ethane temperature used for sample vitrification is increased [*e.g.* to 163 K, (Shi *et al.*, 2019 ▸)]. This is expected since the maximum temperature differences between grid and foil during cooling and thus the maximum strains that must be released are reduced, and because the sample remains at temperatures where water has substantial translational mobility, allowing any sample stress created during cooling to be gradually released until the sample is transferred to LN_2_ and all motion is quenched. Gradually warming samples from ∼90 to 153 K and holding at this temperature for 10 min, or brief heating (5–7 s) to 163 K, both reduce initial beam-induced motion by a factor of 4 (Wieferig *et al.*, 2021 ▸); both allow release of sample stress generated in initial cooling via water diffusion, which leads to partial devitrification and formation of small amounts of cubic ice. These experiments all support the notion that pre-existing compressive stress within the sample drives rapid initial beam induced motion, although they do not identify a unique origin of this compressive stress. An alternative model for the origin of this compressive stress (Naydenova *et al.*, 2020 ▸) makes unphysical assumptions about the maximum stress/pressure that can be generated and sustained in a rapidly cooled aqueous sample, as will be discussed elsewhere.

How might the cooling method and rate affect initial beam-induced motion? The temperature at which a sample transitions from a supercooled liquid to an amorphous ‘solid’, in which subsequent water motions are quenched and stress can be sustained, depends on the cooling rate of the sample. With faster cooling, the time available for motions at and below a given temperature before all motions are quenched decreases and the effective glass transition temperature increases. Water has the character of a strong glass former below ∼170 K, and its translational diffusion coefficient decreases by roughly a factor of 10 for each 10 K temperature drop (Xu *et al.*, 2016 ▸), so that the effective glass transition temperature increases by roughly 10 K for each order of magnitude increase in cooling rate. To minimize stress in the vitrified sample, the effective glass transition temperature should be as low as is feasible, the cooling rate as small as is feasible and the cooling time to the glass transition as long as is feasible, given other constraints. From a less formal perspective, if cryoEM samples never crystallized, cooling as slowly as possible would be the obvious route to minimizing sample stress associated with interaction between and differential contraction of the sample, foil and grid. Since crystallization does occur, cooling rates should then be no larger than is necessary to obtain a largely vitrified sample, having the largest crystalline ice fraction that is compatible with high-resolution imaging and reconstruction. Somewhat smaller cooling rates provided (under ideal circumstances) by boiling nitro­gen than by ethane may then be expected to yield less sample stress and less beam-induced motion.

Note that the density of crystalline ices at *T* = 77 K (∼0.932 g cm^−3^) is slightly less than that of vitreous ice (∼0.944 g cm^−3^) (Loerting *et al.*, 2011 ▸), so one might expect the increase in specific volume on crystallization to create compressive stress in the sample film. However, since ice crystal growth rates drop precipitously below 215 K (Section 2.1), almost all ice must form before the remaining solvent has vitrified, so any local stress associated with ice crystal growth will be released by solvent flow.

Smaller cooling rates may reduce another, related component of sample stress that may drive beam-induced motion. During plunge-cooling, as the grid + sample enters the liquid cryogen, cooling from *T*_m_ ≃ 273 K to *T*_g_ ≃ 136 K occurs within a band on the grid whose width (along the plunge direction) is proportional to the product of the plunge speed and cooling rate. Consequently, increasing the cooling rate reduces the width of this band, which increases the maximum thermal gradient within the band. Non-uniform grid bar contraction due to this thermal gradient creates a trapezoidal deformation of the grid openings and of the foil that spans it, as shown schematically in Fig. S9. Since the foil + sample cools faster than the grid bars, the sample will vitrify on the distorted foil. As cooling continues and the thermal gradient across the grid diminishes, the foil distortion will diminish. This will tend to create a primarily uniaxial compressive stress in the vitrified sample oriented perpendicular to the plunge direction that will again drive radiation-induced creep and beam-induced motion, but not in the radially symmetric way expected if the grid cooled uniformly.

## 6. Conclusions   

The present results establish the feasibility of routine vitrification of single-particle cryo-EM samples using only boiling liquid nitro­gen and show that these samples can yield high-resolution particle reconstructions and refined structures, comparable to those achieved when samples are cooled in liquid ethane. Cooling rates perhaps four times larger (Hua & Xu, 2000 ▸) may be obtained by cooling LN_2_ to just above its freezing temperature (∼63 K) via thermal contact with evaporatively cooled LN_2_. Somewhat larger cooling rates might be achievable using ethane with improved cooling instrument and grid designs. However, single-particle cryo-EM reconstructions are insensitive to small crystalline ice fractions that may result from smaller cooling rates, and slower cooling is likely to reduce sample stresses that drive resolution-limiting beam-induced motion. By eliminating the use of flammable ethane, all-LN_2_ cooling may simplify cryo-EM sample workflows and cold chains and simplify the design of automated sample-cooling instruments, with no compromise in data quality.

## 7. Related literature   

The following reference is cited in the supporting information: Marr *et al.* (2014 ▸).

## Supplementary Material

Supporting figures and tables. DOI: 10.1107/S2052252521008095/eh5013sup1.pdf

## Figures and Tables

**Table 1:** Refinement statistics for an apoferritin structure determined using a 2.64 Å *cryoSPARC* reconstruction, from data collected on a Quantifoil UltraAuFoil grid plunge-cooled in boiling LN_2_ (sample 1)

Data collection		
Microscope	Talos Arctica	
Voltage (kV)	200	
Nominal magnification	63000×	
Exposure navigation		
Cumulative exposure (e^−^ Å^−2^)	55	
Exposure rate (e^−^ pixel^−1^ s^−1^)	28	
Exposure per frame (e^−^ Å^−2^)	1.1	
Detector	K3	
Pixel size (Å)	0.615	
Defocus range (µm)	0.6–1.8	
Micrographs used	136	
Total extracted particles	129019	
Refined particles	95834	
Reconstruction		
Final particles	59824	
Symmetry imposed	Octahedral	
Map sharpening *B*-factor (Å^2^)	124.9	
Resolution (global) (Å)	2.64	
Refinement		
Model composition		
Chains	24	
Atoms	66099 (Hydrogens: 32403)	
Residues	Protein: 4104, nucleotide: 0	
Water	0	
Ligands	0	
Bonds (RMSD)		
Length (Å) (no. > 4σ)	0.006 (0)	
Angle (°) (no. > 4σ)	1.871 (308)	
*MolProbity* score	1.94	
Clash score	10.98	
Ramachandran plot (%)		
Outliers	0.00	
Allowed	5.52	
Favored	94.48	
Rotamer outliers (%)	0.00	
C_β_ outliers (%)	0.00	
Peptide plane (%)		
Cis proline/general	33.3/0.0	
Twisted proline/general	0.0/0.0	
CaBLAM outliers (%)	0.60	
ADP (*B*-factors)		
Iso/Aniso (no.)	33696/0	
Min/max/mean		
Protein	30.00/154.79/133.39	
Nucleotide	–	
Ligand	–	
Water	–	
Occupancy		
Mean	1.00	
Occ = 1 (%)	100.00	
0 < occ < 1 (%)	0.00	
Occ > 1 (%)	0.00	
Box		
Lengths (Å)	128.53, 128.53, 128.53	
Angles (°)	90.00, 90.00, 90.00	
Supplied resolution (Å)	2.6	
Resolution estimates (Å)	Masked	Unmasked
d FSC (half maps; 0.143)	2.7	2.9
d 99 (full/half1/half2)	3.7/1.5/1.5	3.7/1.3/1.3
d model	3.1	3.1
d FSC model (0/0.143/0.5)	–/2.8/3.2	2.6/2.8/3.2
Map min/max/mean	0.18/0.54/0.00	
Model versus data		
*CC* (mask)	0.80	
*CC* (box)	0.84	
*CC* (peaks)	0.72	
*CC* (volume)	0.80	
Mean *CC* for ligands	–	
