# Supplementary material for: High-resolution single-particle cryo-EM of samples vitrified in boiling nitro­gen
Source: IUCrJ. 2021 Sep 7;8(Pt 6):867–77. doi: 10.1107/S2052252521008095 (PMC8562666; doi:10.1107/S2052252521008095)
Supplement: Supplementary file 1 [file m-08-00867-sup1.pdf]

IUCrJ

Volume 8 (2021)

Supporting information for article:

High-resolution single-particle cryo-EM of samples vitrified in boiling nitrogen

Tyler Engstrom, Jonathan A. Clinger, Katherine A. Spoth, Oliver B. Clarke, David S. Closs, Richard Jayne, Benjamin A. Apker and Robert E. Thorne

"*High resolution single-particle cryo-EM using samples vitrified in boiling nitrogen*" by Engstrom *et al.*

S1. Fabrication of grid+foil prototypes

Grid Type B was an in-house developed Cu grid/Au foil prototype. The Cu grid had a 300 mesh hexagonal pattern and was electroformed to a 15 μm thickness using the vertically profiled grid (VPG) technique. The gold foil was made by thermally evaporating a 35 nm thick release layer of sodium metaphosphate (its precursor is available as Victawet 35B from Ladd Research) on an e-beam lithography master with a 1.2 μm/1.3 μm hole pattern (Toronto Nanofabrication Centre), and then evaporating 35 nm of gold on top of the release layer. Foils were floated off the master by dipping at a shallow angle in DI water, grids were applied to floating foils, and the grid-foil assemblies lifted off with Parafilm backing paper, following a method used for Formvar films (Marr *et al.*, 2014). Assembled grids were allowed to dry overnight and inspected both optically and in an SEM. Evaporation and SEM imaging were performed at Cornell's Center for Materials Research.

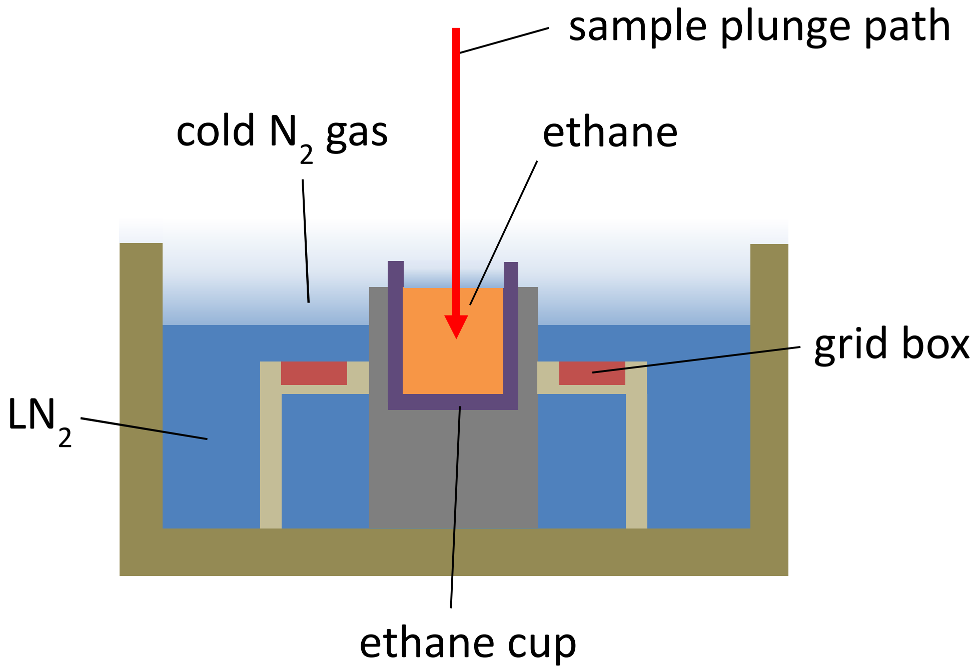

Figure S1 A typical set-up used for sample cooling in single-particle cryo-EM. Liquid ethane at T ~90 K is held in a small metal cup that is in weak thermal contact with boiling LN_2 at 77 K held within a surrounding insulated container. Cold gas (primarily N_2) is generated by boiling of the LN_2 and by direct thermal conduction from the cold ethane and LN_2 surfaces. The grid and especially the foil and sample between grid openings are cooled as they pass through this cold gas toward the ethane surface.

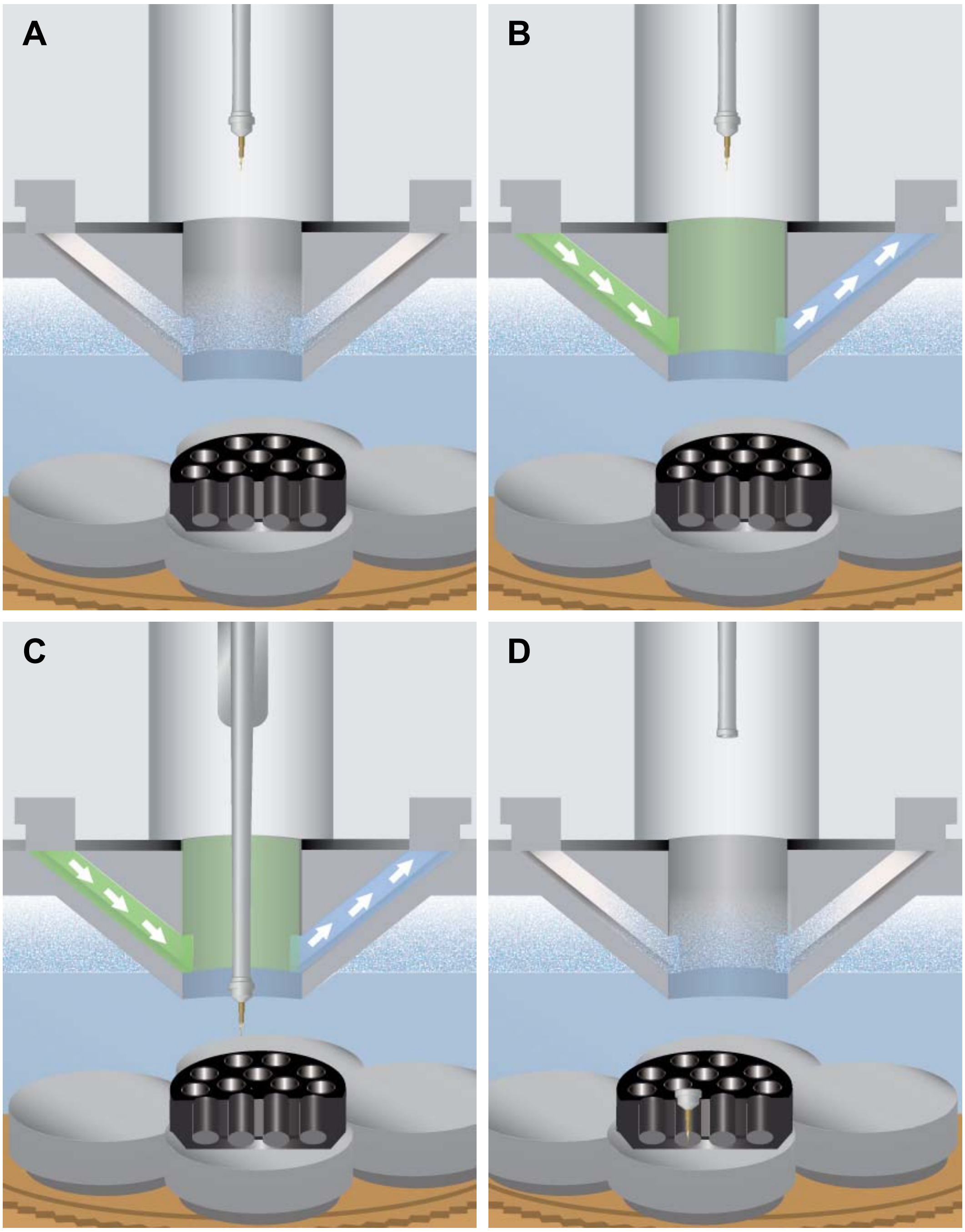

Figure S2 Schematic illustration of a sample plunge sequence in the NANUQ automated LN_2-based plunge cooler. **A** Cold gas is initially present in the plunge bore above the LN_2 surface. **B** At the start of the plunge sequence, the cold gas is removed and replaced with ambient temperature dry N_2 gas. **C** The sample is plunged at 2 m/s into the LN_2. All cooling occurs in the LN_2, with little or no precooling in cold gas. **D** The sample is then translated into a crystallography "puck" that holds 16 samples. Either a UniPuck (shown) or a CombiPuck (in which samples are inserted into cryovials held within the puck) can be used.

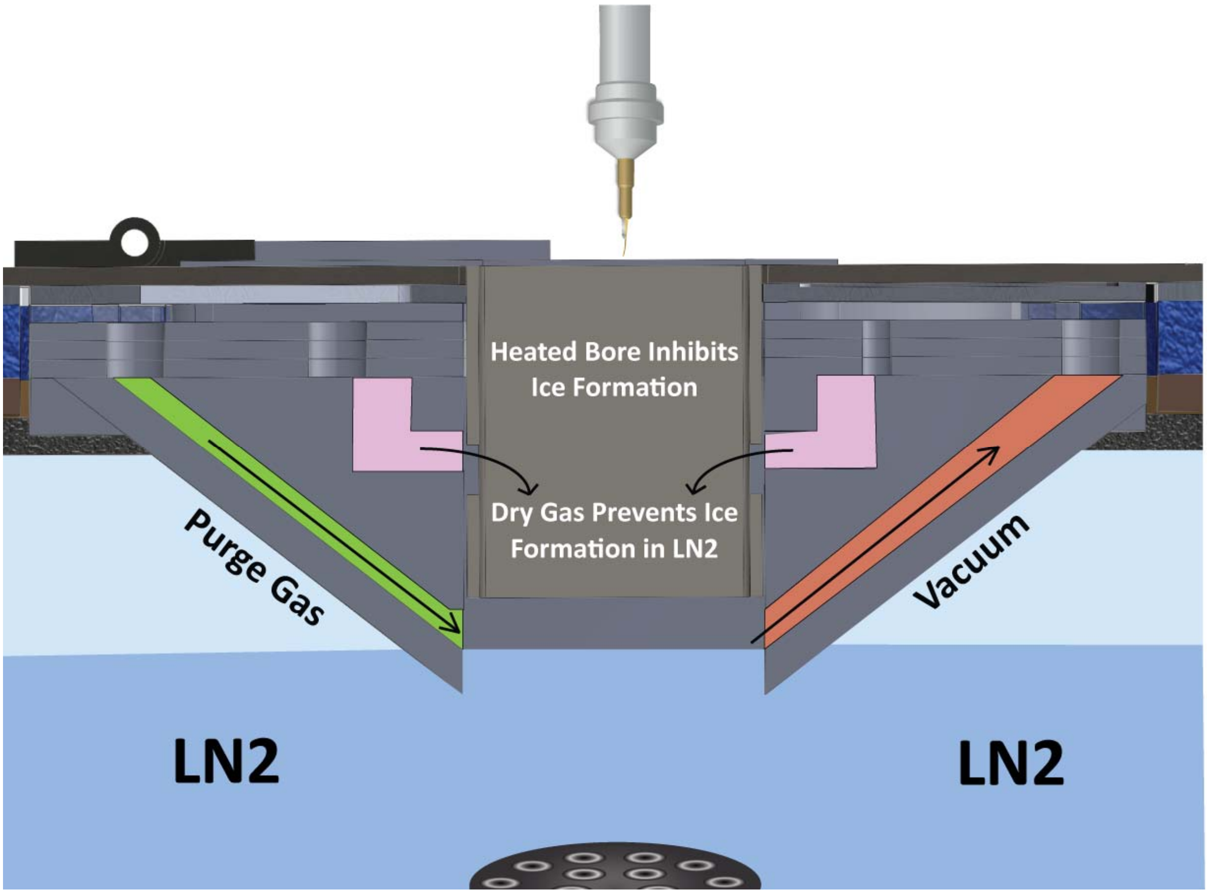

Figure S3 The gas management manifold of the NANUQ automated LN_2-based plunge cooler. The manifold isolates the LN_2 surface within the plunge bore. Vacuum removes cold gas and is replaced by ambient temperature make-up gas that sweeps across the LN_2 surface. Additional ambient temperature gas floods the bore to isolate it from moist ambient air, and heaters maintain the bore walls at room temperature. The LN_2 level is precisely maintained as shown using an automated level control system. This system both eliminates all sample precooling in cold gas and completely isolates the LN_2 and other cold surfaces from moist ambient air, preventing frosting.

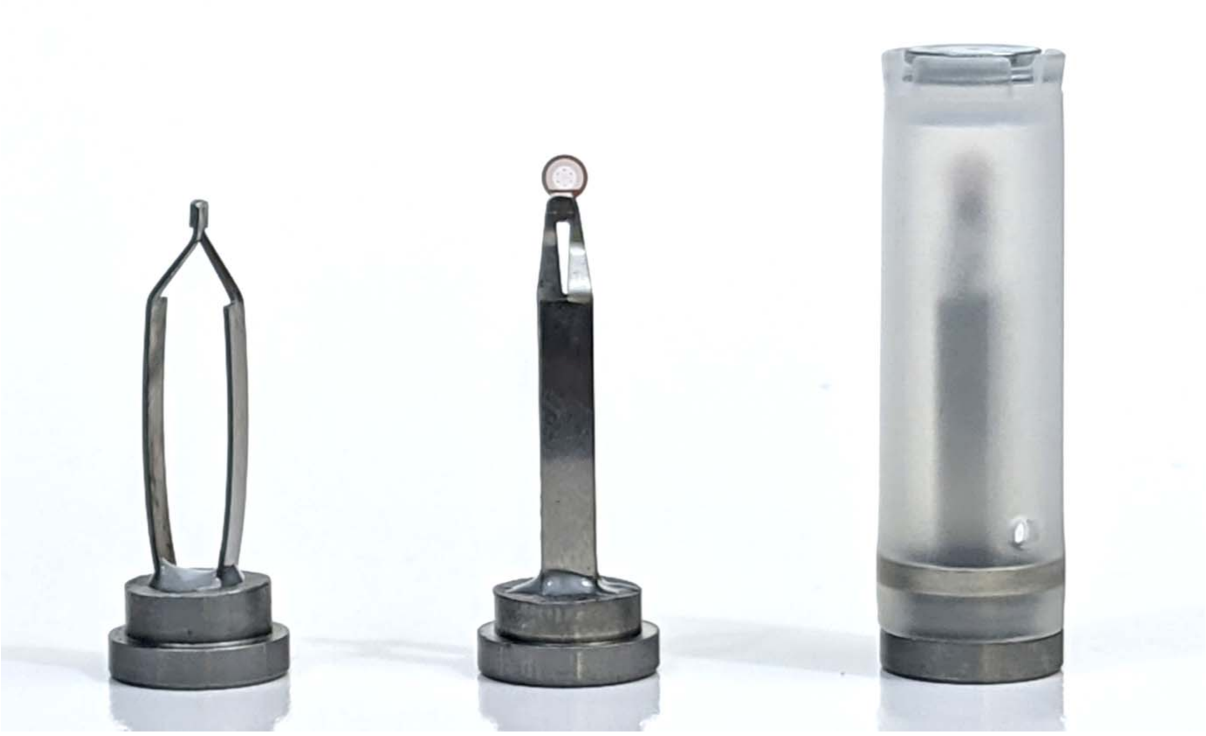

Figure S4 Grid gripping forceps integrated into a standard crystallography goniometer base. The bottom of the base attaches to a standard magnetic crystallography wand and to the magnetic wand used to hold samples in Nanuq's plunge stage. After plunging, the grid, forceps and base are stored in a cryovial-containing CombiPuck.

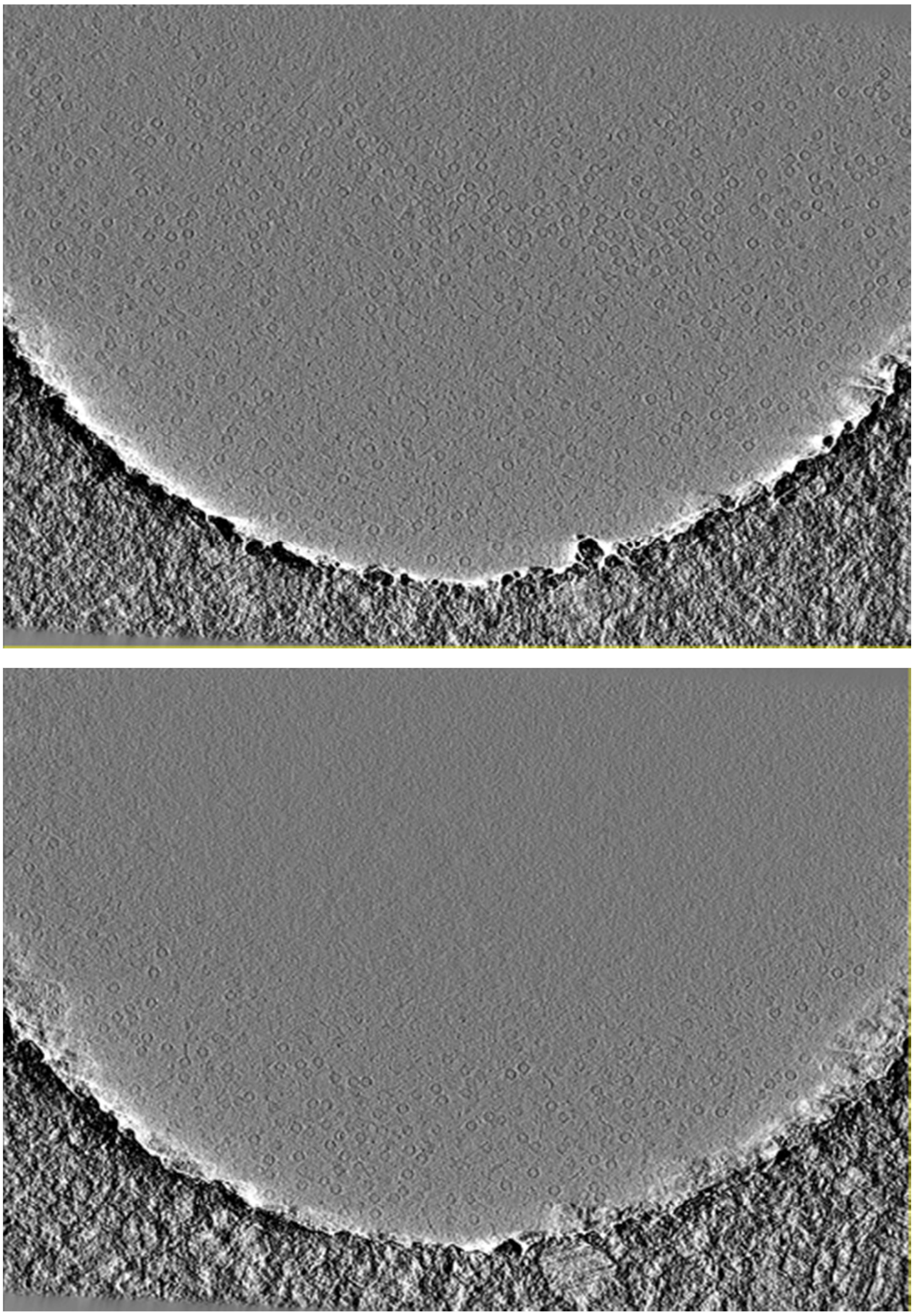

Figure S5 A tomogram slice through the middle of a film spanning a 1.2 μm hole in Sample 1, cooled with LN_2, showing an approximately uniform apoferritin particle distribution within this plane. **B** Another slice from the same tomogram, but shifted 20 nm (16 pixels) in the out-of-plane direction, showing how thicker ice around the hole edges can accommodate an extra layer of apoferritin.

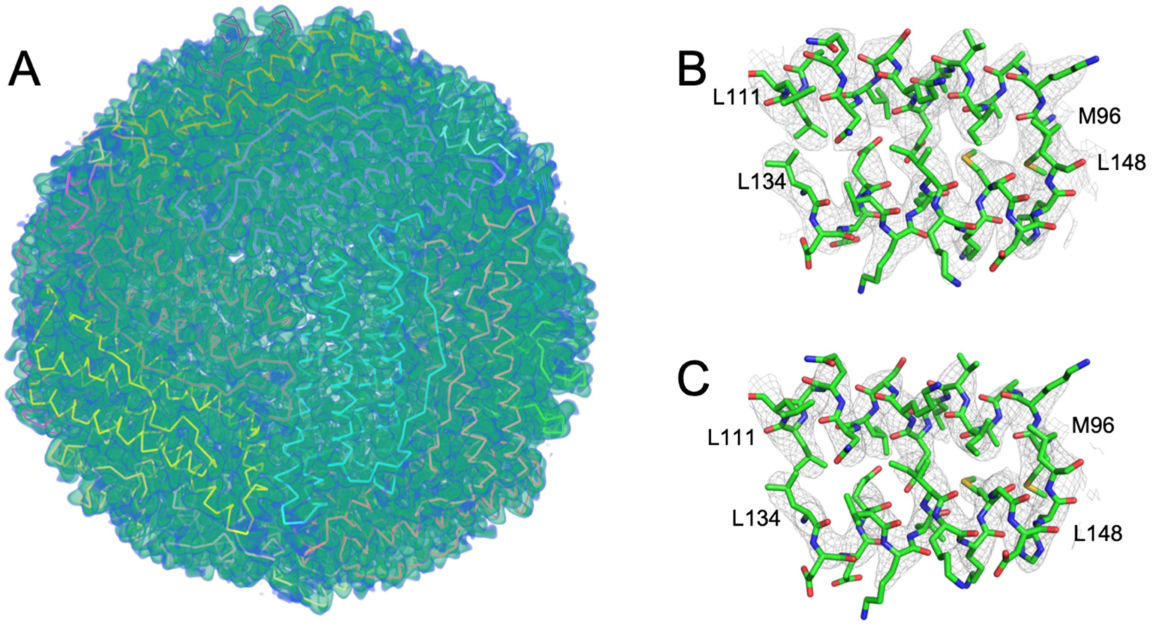

Figure S6 Single-particle reconstruction and refined model based on apoferritin data obtained from Sample 2, which was deposited on a MiTeGen prototype grid and plunge cooled in LN_2. **A** Apoferritin model placed into the reconstructed electron density map. Ribbons of apoferritin monomers are colored by chain designation. The map is colored by σ value, with green and blue corresponding to 2σ and 1σ, respectively. **B** Portion of chain A zoomed in to illustrate model fit to the map. The map mesh (gray) is contoured at 1σ. **C** The same portion of chain A as in **B** with mesh contoured at 2σ. Fourier shell correlation (FSC) plot is shown in SI Fig. S7(b).

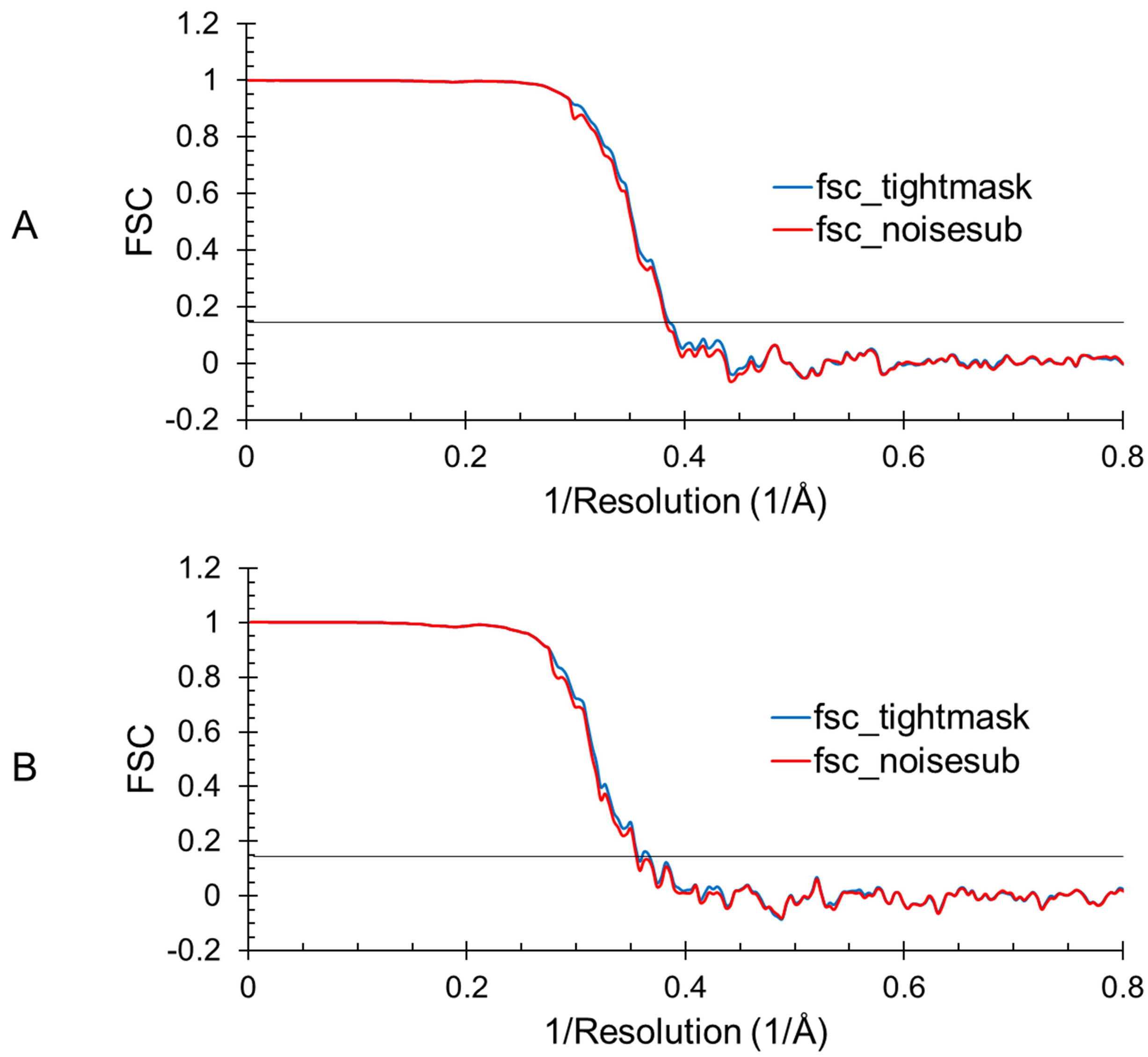

Figure S7 Fourier shell correlation (FSC) plots for **A** Sample 1 and **B** Sample 2, determined using Cryosparc.

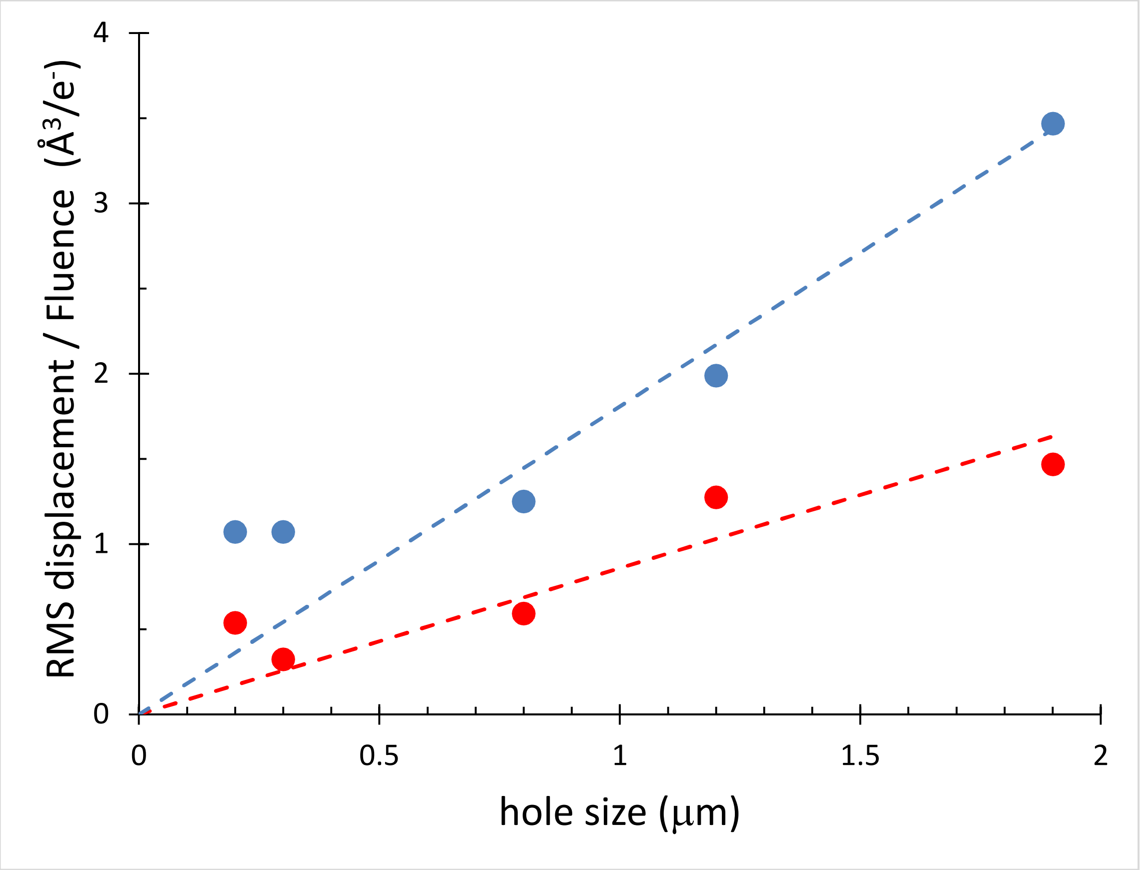

Figure S8 RMS local particle displacement per unit fluence versus foil hole size, determined from Fig. 1 of Naydenova et al. using the first measured data point (blue) and at a fluence of 2.5 $e^{-}/Å^{2}$. Particle displacement increases linearly with hole size. Naydenova et al. does not have multiple measurements in the low dose region where the displacement increases most rapidly, so the values given here assume the initial slope of the displacement vs fluence data passes through zero fluence. Actual initial slopes of displacement vs fluence may be somewhat larger.

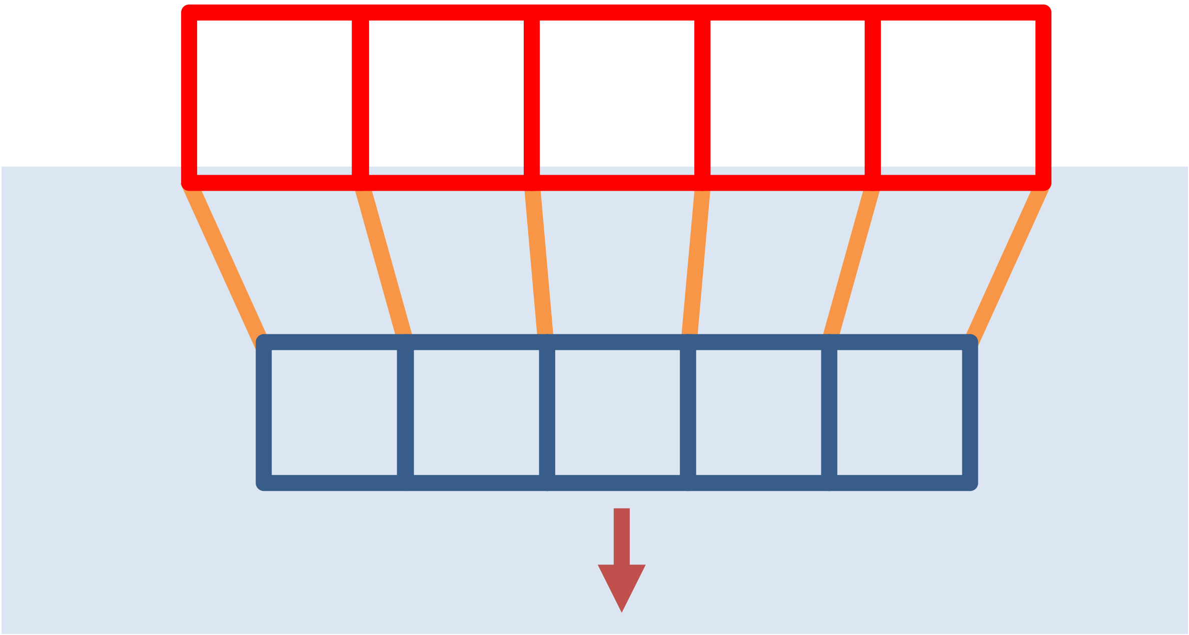

Figure S9 Schematic illustration of transient grid deformation during plunge cooling due to transient temperature gradients that develop as the grid enters the liquid cryogen.

Table S1 Refinement statistics for an apoferritin structure determined using a 2.86 Å cryoSPARC reconstruction. CryoEM data was obtained from Sample 2, which was placed on a MiTeGen prototype grid and plunge cooled in boiling LN_2. Over 90% of hole images and image FFTs showed no evidence of crystalline ice, indicating near complete vitrification.

**Data collection**	
Microscope	Talos Arctica
Voltage (kV)	200
Nominal magnification	63,000×
Exposure navigation	
Cumulative exposure (e^{-} $Å^{-2}$)	55
Exposure rate (e^{-} $pixel^{-1}$ s^{-1})	28
Exposure per frame (e^{-} $Å^{-2}$)	1.1
Detector	K3
Pixel size (Å)	0.615
Defocus range (μm)	0.5-1.7
Micrographs used	122
Total extracted particles (no)	109,901
Refined particles (no)	84,369
**Reconstruction**	
Final particles (no)	22,027
Symmetry imposed	octahedral
Map Sharpening B factor ($Å^2$)	118.5
Resolution (global) (Å)	2.85
**Refinement**	
Model Composition (#)	
Chains	24
Atoms	32736 (Hydrogens: 0)
Residues	Protein: 4080 Nucleotide: 0
Water	0
Ligands	0
Bonds (RMSD)	
Length (Å) (# >4σ)	0.006 (0)
Angles (°) (# >4σ)	1.118 (53)
MolProbity score	1.68
Clash score	9.64
Ramachandran plot (%)	
Outliers	0.00
Allowed	3.03
Favored	96.97
Rotamer outliers (%)	0.03
C_β outliers (%)	0.05
Peptide plane (%)	
Cis proline/general	0.0/0.0
Twisted proline/general	0.0/0.0
CaBLAM outliers (%)	1.15
ADP (B-factors)	
Iso/Aniso (#)	32736/0

min/max/mean		
Protein	112.88/156.90/130.13	
Nucleotide	---	
Ligand	---	
Water	---	
Occupancy		
Mean	1.00	
occ = 1 (%)	100.00	
0 < occ < 1 (%)	0.00	
occ > 1 (%)	0.00	
Data		
Box		
Lengths (Å)	130.38, 129.76, 130.38	
Angles (°)	90.00, 90.00, 90.00	
Supplied Resolution (Å)	2.8	
Resolution Estimates (Å)	Masked	Unmasked
d FSC (half maps; 0.143)	2.8	2.9
d 99 (full/half1/half2)	3.9/1.4/1.4	3.9/1.3/1.3
d model	3.2	3.2
d FSC model (0/0.143/0.5)	2.7/2.8/3.4	2.7/2.9/3.6
Map min/max/mean	-0.13/0.48/0.04	
Model vs. Data		
CC (mask)	0.83	
CC (box)	0.89	
CC (peaks)	0.77	
CC (volume)	0.82	

Table S2 Motion statistics generated from positive net x-y displacements (uniform and nonuniform components) over 50 frames, for Sample 1 on a Quantifoil UltrAuFoil (Type A) grid and Sample 2 on a prototype grid (Type B, gold foil on copper grid) plunge cooled in LN_2 and for a Quantifoil UltrAuFoil grid (Type A) plunge cooled in ethane using a Vitrobot Mark IV (courtesy of Ryan Feathers), all measured using the same microscope. First and second numbers in the bottom two rows are mean and standard deviation, respectively. Fig. 5 shows the corresponding local motion data versus fluence.

**Grid type / Cooling method**	**Type A / LN_2**	**Type B / LN_2**	**Type A / Ethane**
Number of images	160	174	450
Number of picked particles	129,019	109,901	151,621
Total dose over 50 frames [e^-/ $Å^2$]	54.5	54.5	40
Full frame rigid motion [Å]	33 ± 74	22 ± 48	32 ± 74
Full frame local motion [Å]	16 ± 25	8.4 ± 13	29 ± 66
